# Replication kinetics and infectivity of SARS-CoV-2 variants of concern in common cell culture models

**DOI:** 10.1186/s12985-022-01802-5

**Published:** 2022-04-26

**Authors:** Lena Mautner, Mona Hoyos, Alexandra Dangel, Carola Berger, Anja Ehrhardt, Armin Baiker

**Affiliations:** 1 Unit of Molecular Biologic Analytics and Biogenetics, Bavarian Health and Food Safety Authority, Veterinaerstrasse 2, 85764 Oberschleißheim, Germany; 2 Public Health Microbiology Unit, Bavarian Health and Food Safety Authority, 85764 Oberschleißheim, Germany; 3 Virology and Microbiology, Center for Biomedical Education and Research (ZBAF), Witten/Herdecke University, 58453 Witten, Germany

**Keywords:** SARS-CoV-2, VOC, Kinetics, Omicron, Cell culture, Caco-2, Calu-3

## Abstract

**Background:**

During the ongoing Covid-19 pandemic caused by the emerging virus SARS-CoV-2, research in the field of coronaviruses has expanded tremendously. The genome of SARS-CoV-2 has rapidly acquired numerous mutations, giving rise to several Variants of Concern (VOCs) with altered epidemiological, immunological, and pathogenic properties.

**Methods:**

As cell culture models are important tools to study viruses, we investigated replication kinetics and infectivity of SARS-CoV-2 in the African Green Monkey-derived Vero E6 kidney cell line and the two human cell lines Caco-2, a colon epithelial carcinoma cell line, and the airway epithelial carcinoma cell line Calu-3. We assessed viral RNA copy numbers and infectivity of viral particles in cell culture supernatants at different time points ranging from 2 to 96 h post-infection.

**Results:**

We here describe a systematic comparison of growth kinetics of the five SARS-CoV-2 VOCs Alpha/B.1.1.7, Beta/B.1.351, Gamma/P.1, Delta/B.1.617.2, and Omicron/B.1.1.529 and a non-VOC/B.1.1 strain on three different cell lines to provide profound information on the differential behaviour of VOCs in different cell lines for researchers worldwide. We show distinct differences in viral replication kinetics of the SARS-CoV-2 non-VOC and five VOCs on the three cell culture models Vero E6, Caco-2, and Calu-3.

**Conclusion:**

This is the first systematic comparison of all SARS-CoV-2 VOCs on three different cell culture models. This data provides support for researchers worldwide in their experimental design for work on SARS-CoV-2. It is recommended to perform virus isolation and propagation on Vero E6 while infection studies or drug screening and antibody-based assays should rather be conducted on the human cell lines Caco-2 and Calu-3.

**Supplementary Information:**

The online version contains supplementary material available at 10.1186/s12985-022-01802-5.

## Introduction

The novel severe acute respiratory syndrome coronavirus 2 (SARS-CoV-2) was identified in Wuhan, China, in late 2019 and declared a pandemic by the World Health Organization (WHO) in March 2020 [1, 2]. Despite encoding a proofreading function, the SARS-CoV-2 genome has acquired numerous mutations during the worldwide spread of the virus [3]. The initially circulating Wuhan strain was rapidly replaced by a variant containing the D614G mutation in its spike protein, which is associated with higher viral loads in patients and increased infectivity [4, 5]. From late 2020 on, several new variants emerged. They exhibited various new epidemiological, immunological or pathogenic properties. These variants are constantly monitored by the WHO and national health authorities as Variants of Concern (VOCs) or Variants under Investigation (VUIs). The first VOC, designated Alpha/B.1.1.7 and initially detected in the UK, showed a higher transmissibility compared to both the original Wuhan strain and the D614G variant [6–8]. It became dominant during the third Covid-19 wave in winter 2020/21 in most European and North American countries. While the Alpha variant remains mostly susceptible to neutralization by the immune system, both variants Beta/B.1.351 and Gamma/P.1 (first detected in South Africa and Brazil, respectively) escape from neutralizing antibodies in patient sera, threatening the success of vaccination efforts [9–11]. Shortly after, the pandemic was dominated by yet another VOC named Delta/B.1.617.2, which has rapidly displaced other variants worldwide since its emergence in India in April 2021 [12]. Its ubiquitous spread is attributed to a combination of increased fitness and moderate immune escape [13, 14]. On November 26, 2021, WHO designated a new variant named Omicron/B.1.1.529 as the fifth VOC. This variant immediately raised global concerns due to its numerous mutations compared to the other VOCs. Relative to Delta, Omicron is highly mutated with at least 30 amino acid substitutions in the spike protein and 15 of these residing in the receptor-binding domain (RBD) [15, 16]. Importantly, these mutations related to Omicron may be linked to increased transmissibility, stronger viral binding affinity, and antibody escape. Omicron mutations with documented consequences include those that improve transmissibility and alter binding affinity [17–19].

Cell culture models are important tools to study viral replication and tropism, to identify potential drug targets and to test antiviral compounds [20–22]. The African Green Monkey-derived Vero E6 kidney cell line is widely used in coronavirus research for virus stock propagation and antiviral assays [23, 24]. However, while it does express the ACE2 receptor for SARS-CoV-2 attachment, it lacks the TMPRSS2 protease required for entry into human cells [25–27]. Instead, viral entry into Vero E6 is likely cathepsin-mediated and may not accurately mimic the infection event in human cells [28–30]. Human cell models include the colon epithelial carcinoma cell line Caco-2 and the airway epithelial carcinoma cell line Calu-3. Expressing both ACE2 and TMPRSS2, these cell lines are highly permissive for SARS-CoV-2 infection and have been employed in thorough virus characterization [22, 25, 26, 31, 32].

Epidemiological data on differences in VOC transmissibility raises the question, if cell culture models can adequately reflect these variations. However, so far studies of VOC performance in cell culture are limited to pseudo-typed viruses or incomplete observations of individual VOCs on single cell lines [13, 33–36]. Differences in experimental settings impede cross-comparison of multiple studies. To obtain a comprehensive picture, we systematically assessed replication kinetics and infectivity of a non-VOC strain and the five VOCs Alpha, Beta, Gamma, Delta, and Omicron on the three widely used cell lines Vero E6, Caco-2, and Calu-3. Cells were infected for a multi-step growth curve with an equivalent, non-saturating multiplicity of infection (MOI) of 0.0001 for 96 h to represent typical propagation experiments with the VOCs under standard laboratory conditions and culture supernatants were used for virus quantification by RT-qPCR and determination of infectious particles.

## Materials and methods

### Cell culture

African Green monkey kidney cells Vero E6 (ATCC^®^ CRL-1586™) were grown in DMEM growth medium supplemented with 10% heat-inactivated fetal calf serum (FCS; Gibco, Invitrogen, Carlsbad, USA) and 1% penicillin–streptomycin solution (10,000 U/mL, Gibco). Human colorectal adenocarcinoma cells Caco-2 and human airway epithelial cells Calu-3 were kindly donated by Dr. Ulrich Lächelt from the Department of Pharmacy at the Ludwig-Maximilians-University Munich. They were cultured in EMEM growth medium supplemented with 10% (Calu-3) or 20% FCS (Caco-2) and 1% penicillin–streptomycin solution. All cells were maintained at 37 °C in a humidified atmosphere in the presence of 5% CO_2_. All cell lines were free of mycoplasma contaminations as tested by PCR. The authenticity of the used human cell lines was determined by SNP typing.

### Isolation and propagation of clinical SARS-CoV-2 strains

Experiments with SARS-CoV-2 were performed in a BSL-3 laboratory following applicable safety and security protocols. Clinical SARS-CoV-2 strains non-VOC/B.1.1, Alpha/B.1.1.7, Beta/B.1.351, Gamma/P.1, Delta/B.1.617.2, and Omicron/B.1.1.529 were obtained from patients with laboratory-confirmed diagnosis of SARS-CoV-2 infection as previously described [37]. Briefly, the patients’ pharyngeal swab samples were filtered through a 0.45 μm Minisart^®^ syringe filter (Sartorius Stedim Biotech, Goettingen, Germany) and inoculated on a monolayer of Vero E6 cells until the typical cytopathic effect (CPE) was visible. After verifying the integrity of the SARS-CoV-2 isolate in the cell culture supernatant by RT-qPCR utilizing the Xpert^®^ Xpress SARS-CoV-2 (#XPRSARS-COV2-10, Cepheid, Sunnyvale, CA, USA), the isolates were further propagated on Vero E6 cells. Viral stocks were generated from infected cell culture supernatants and stored at – 80 °C until further usage. The viruses were sequenced by whole-genome sequencing (WGS) as described in [38] directly from the clinical samples and again upon virus isolation. For all non-VOC, Alpha, Beta, Gamma, and Delta samples the artic primer scheme V3 was used. Passage 1 and 2 of Omicron were sequenced using primer scheme V4, while for the replicates of passage 3 the V4.1 primers were employed (for gisaid database (www.gisaid.org) accession numbers, see paragraph ‘Availability of data and materials’). Titration of infectious particles of viral stocks was performed on Vero E6 by end-point dilution assay determining 50% tissue culture infectious dose (TCID50).

### Infection experiments

Vero E6, Caco-2, and Calu-3 cells were seeded 1–5 days prior to infection in T-75 cell culture flasks. Before infection, cells of one representative T-75 cell culture flask per cell line were detached with trypsin/EDTA and counted. Cell lines were infected with the SARS-CoV-2 isolates non-VOC/B.1.1, Alpha/B.1.1.7, Beta/B.1.351, Gamma/P.1, Delta/B.1.617.2, and Omicron/B.1.1.529 in triplicates (n = 3) at an MOI of 0.0001 in 4 mL of their respective cell culture media for 2 h. Cells were washed twice with PBS and reconstituted with 12 mL of their respective cell culture media. At the time points 2 h (directly after reconstitution in cell culture media) as well as 8, 24, 32, 48, 56, 72, 80, and 96 h post-infection (p.i.), samples were taken from the supernatant. Simultaneously, microscopy pictures were generated to observe the development of CPE.

### Analysis of viral particle release by RT-qPCR

For quantification of extracellular viral RNA, supernatants were heat-inactivated for 90 min at 65 °C. Quantitative reverse transcription real-time polymerase chain reaction (RT-qPCR) analysis was performed using FTD SARS-CoV-2 (#FTD-114–96, Fast Track Diagnostics, Eschsur-Alzette, Luxembourg) after RNA extraction utilising a Maxwell 48 extraction robot with the Maxwell^®^ RSC Blood DNA Kit (#AS1400, Promega GmbH, Mannheim, Germany). Reactions were performed in accordance to the manufacturer’s instructions and carried out in a QuantStudio 7 real-time thermal cycler (Thermo Fisher Scientific, Waltham, USA). QuantStudio™ Real-Time PCR software (Thermo Fisher Scientific) was used for data acquisition and analysis. A standard curve for quantification of genome copy numbers was performed in parallel using purified SARS-CoV-2 viral RNA with genome copy numbers assessed by reverse transcriptase droplet digital PCR (RT-ddPCR) as previously described [37].

### Analysis of infectious viral release by end-point dilution assay

Release of infectious viral particles into the supernatant was assessed using Vero E6 cells cultured in 96-well plates. A tenfold dilution series from each sample was used to infect six independent cell culture wells for determination of inoculation titers and at least four replicate culture wells for experiment read out samples. The CPE was observed under a light microscope at day 6 post-infection (p.i.), and each well was scored either positive or negative for virus infection. An improved Spearman-Kärber method was used to calculate the results, which are presented as TCID50/mL [39, 40].

## Results

We used three relevant cell culture models, Vero E6, Caco-2, and Calu-3, to analyse the replication kinetics of the SARS-CoV-2 VOC strains Alpha, Beta, Gamma, Delta, and Omicron, which had been isolated in Munich, Germany, between January and December 2021. For comparison, we also included a non-VOC strain (B.1.1) isolated in March 2020, which already contained the spike D614G mutation. Low passage virus stocks were used for all virus variants (P1 to P3). To obtain comparable virus stocks for all infection experiments, we passaged the six virus strains once more on Vero E6 cells using equivalent TCID50 doses corresponding approximately to an MOI of 0.001. After 72 h, cell culture supernatants containing infectious virus particles were collected and titrated on Vero E6. These supernatants were subsequently used to start the infection experiments on Vero E6, Caco-2, and Calu-3 cells with a very low MOI of 0.0001 to allow for a multi-step growth curve representative of typical laboratory propagation experiments with extended observation time. It has to be noted that the virus stock production on Vero E6 had resulted in varying ratios between genome copy numbers and the number of infectious particles for the different virus strains. While the ratios for Alpha, Beta, and Gamma were comparable, tenfold less infectious particles for the same genome copy number were obtained for Delta and Omicron, but tenfold more for the non-VOC strain, respectively. This is reflected in variations of the genome copy numbers obtained at the start of the infection experiments, as these were started with a fixed number of infectious units.

**Fig. 1:**
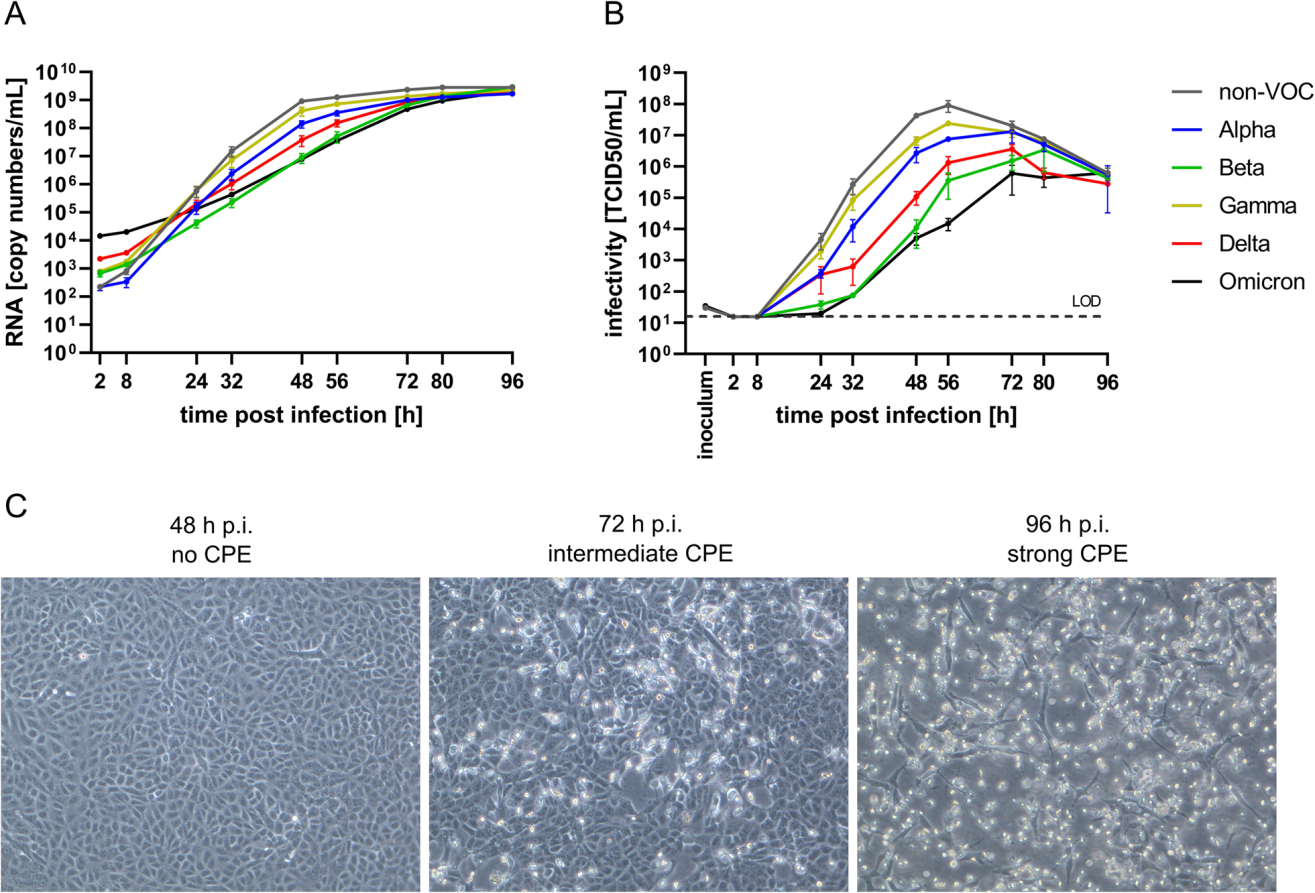
Viral replication of non-VOC and VOCs on Vero E6. Cells were infected at an MOI of 0.0001 for 96 h and culture supernatant was collected at the indicated time points to quantify **a** RNA copy numbers by RT-qPCR and **b** viral titers by TCID50 endpoint assay. **c** Development of CPE is exemplarily shown for Delta at selected time points. LOD: limit of detection; n = 3

On Vero E6 cells, RNA copy numbers detected in the supernatant rapidly rose within the first 48 h p.i. before they started to plateau around 72 h p.i. (Fig. 1a). While individual kinetics differed slightly between the tested virus variants, all variants eventually reached similar RNA copy numbers at the end of the experiment 96 h p.i.. The non-VOC strain showed the fastest replication, gradually followed by the VOCs Gamma, Alpha, Delta, Beta, and Omicron. The maximum difference in RNA copy numbers at a given time point between the non-VOC and Omicron as the most slowly replicating VOC was almost 100-fold at 48 h p.i.. The gradual disparity of RNA copy number kinetics was reflected well in the analysis of viral titers over time (Fig. 1b). The number of infectious viral particles increased most quickly for the non-VOC strain, expeditiously rising to a maximum titer of almost 10^8^ TCID50/mL, again gradually followed by Gamma, Alpha, Delta, Beta, and Omicron. At 56 h p.i., the difference in viral titer spanned even four orders of magnitude between non-VOC and Omicron. In contrast to RNA copy numbers, no plateau but rather a peak in infectious particle release could be observed at 56–72 h p.i. before the titers started to decrease while RNA levels still increased. Comparing the peak infectivity for all strains, there was still almost a 500-fold difference between the highest (non-VOC at 56 h p.i.) and lowest (Omicron at 72 h p.i.) peak in viral titer. In addition to genome copy numbers and infectious viral particle release kinetics, we also recorded the development of the distinct CPE by light microscopy at each time point. The observed CPE in Vero E6 cells appeared rather uniform in evenly spread rounding and sloughing of cells throughout the cell culture flask. Over time, an increasing number of cells rounded, sloughed, and finally lysed and detached from the surface of the cell culture flask, leaving only a few adherent cells (Fig. 1c and Additional file 2: Fig. S1). At the very low MOI used, for non-VOC and VOCs Alpha, Gamma, and Delta, CPE started to show only at 56 h p.i., when RNA copy number levels already started to saturate and the peak of viral titer was reached. For the VOCs Beta and Omicron CPE started even later at only 72–80 h p.i.. Thus, the time point of CPE beginning to show correlated well with the time point of the peak in infectious viral titers in the supernatant, for each viral strain respectively. The most productive phase of infectious viral particle release in Vero E6 cells apparently proceeded without any obviously detectable cell damage. CPE became visible only when the number of infectious viral particles in the supernatant started to decrease.

Because of the widely discussed possibility of mutations at the furin cleavage site when propagating SARS-CoV-2 on Vero E6, we sequenced our virus preparations from patient samples throughout passaging and at the end of this 96 h experiment. Especially analysing the much-questioned furin cleavage site, we could not detect any developing mutations in our SARS-CoV-2 strains upon passaging on Vero E6. The sequence of the furin cleavage site was identical in all samples after 96 h propagation on Vero E6 cells and in the respective previous passages and patient swab samples.

**Fig. 2:**
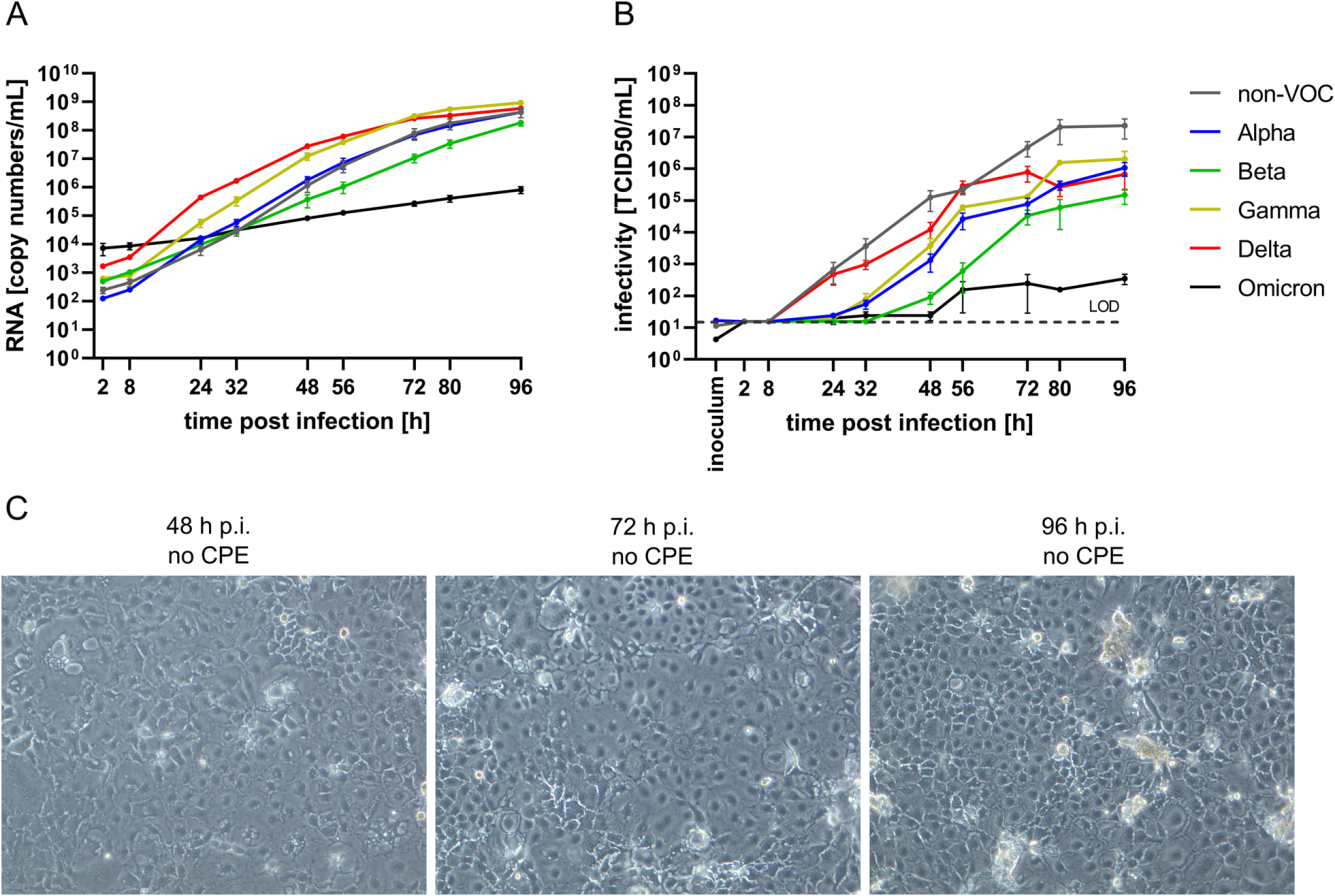
Viral replication of non-VOC and VOCs on Caco-2. Cells were infected at an MOI of 0.0001 for 96 h and culture supernatant was collected at the indicated time points to quantify **a** RNA copy numbers by RT-qPCR and **b** viral titers by TCID50 endpoint assay. **c** Lack of clear CPE is exemplarily shown for Delta at selected time points. LOD: limit of detection; n = 3

On Caco-2 cells, the increase in RNA copy numbers was less steep than on Vero E6 and the RNA copy numbers were approximately tenfold lower than on Vero E6 cells (Fig. 2a). However, RNA levels in the supernatant had apparently not completely saturated yet at 96 h p.i. when the last samples were collected, indicating that maximum virus production had not yet been reached. While Beta again showed slow replication, Delta and Gamma seemed to replicate more efficiently on Caco-2 than the other variants and the non-VOC strain, although differences in RNA copy numbers at the start of the experiment have to be considered. The currently circulating VOC Omicron showed an impaired replication in Caco-2 cells as compared to all other variants, with its genome copy numbers barely increasing by 100-fold within 96 h. Notably, Omicron replication on Vero E6 cells was not as tremendously impaired. Similar to RNA levels, the viral titers had apparently not saturated yet, but kept increasing until 96 h p.i. (Fig. 2b). In contrast to the results from Vero E6, there was a larger discrepancy between RNA levels and viral titers on Caco-2 cells. While the non-VOC strain showed only intermediate RNA copy numbers, its titers exceeded those of all VOCs at almost all time points. Interestingly, the Alpha variant replicated very similarly to the non-VOC in terms of RNA copy numbers, but exhibited considerably lower infectious particle titers. Within VOCs, the general trend was similar between RNA levels and infectious particles with Delta and Gamma showing the fastest replication, followed by Alpha and Beta and leaving Omicron solely far behind. Regarding viral titers in cell culture supernatant, there was a strong difference in TCID50/mL of almost six orders of magnitude between the overall highest titer (non-VOC at 80/96 h p.i.) and the highest titer Omicron reached after 96 h p.i.. A striking difference to Vero E6 was the lack of a clear CPE on Caco-2 (Fig. 2c). There were slight morphological changes detectable and some small areas in the cell layer showed distinct cell damage, but overall cells kept dividing and remained healthy. This CPE absence cannot be attributed to a premature end of the experiment, as it has been reported previously [41] and was observable as well during prolonged virus infection over multiple weeks (data not shown).

**Fig. 3:**
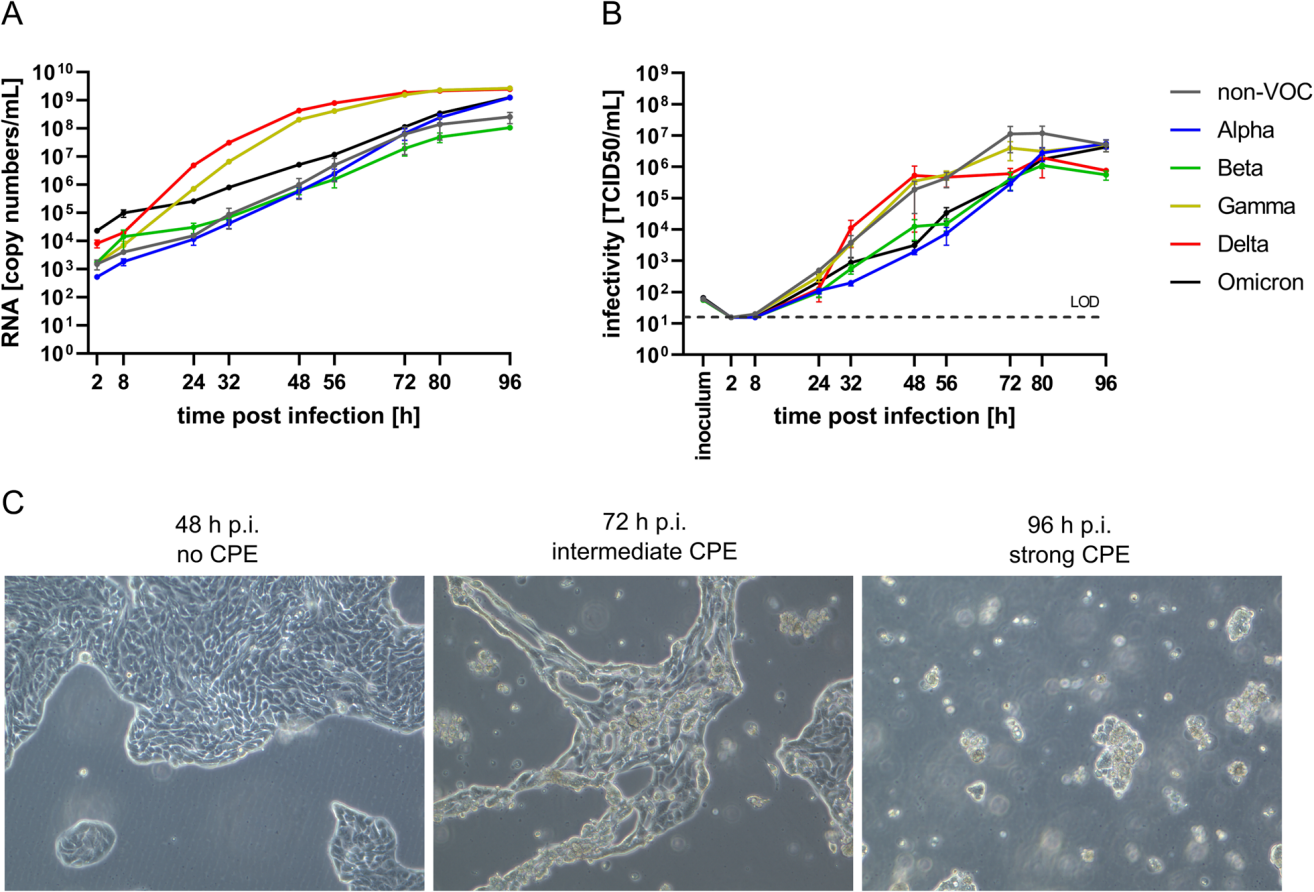
Viral replication of non-VOC and VOCs on Calu-3. Cells were infected at an MOI of 0.0001 for 96 h and culture supernatant was collected at the indicated time points to quantify **a** RNA copy numbers by RT-qPCR and **b** viral titers by TCID50 endpoint assay. **c** Development of CPE is exemplarily shown for Delta at selected time points. LOD: limit of detection; n = 3

Infection kinetics on Calu-3 cells resembled those on Caco-2 besides clearly faster replication kinetics of Delta and Gamma and a less dramatic replication disadvantage of Omicron. RNA copy numbers of non-VOC, Alpha and Beta increased relatively slow compared to on Vero E6 cells and were probably not fully saturated yet at 96 h p.i. (Fig. 3a). In contrast, RNA levels for Delta and Gamma rose rapidly and plateaued around 72 h p.i., with the maximum difference between Delta and Beta being more than 1.000-fold at 56 h p.i.. Omicron showed an intermediate replication capacity on Calu-3 cells and a clearly slower increase in RNA copy numbers than the variants Gamma and Delta, but slightly higher numbers than non-VOC, Alpha, and Beta. Viral titers seemed to peak close to the end of the experiment at 80 h p.i.. Regarding Omicron, its viral titers on Calu-3 were more comparable to those of the VOCs Alpha and Beta than to the more recently circulating VOC Delta. Despite comparability of Omicron in terms of RNA copy numbers and viral titers in cell culture supernatant, the development of CPE on Calu-3 cells was outstandingly slow and weak for Omicron (Additional file 4: Fig. S3). Of note, infection of Calu-3 cells with Delta led to obvious CPE already after 56 h p.i., gradually followed by Gamma, Alpha, and non-VOC between 72 and 80 h p.i. and the VOC Beta showing clear CPE at least at 96 h p.i.. Infection with the currently rapidly spreading VOC Omicron did not lead to a definite CPE until the end of the experiment at 96 h p.i. (Additional file 4: Fig. S3).

A summary of peak RNA levels and infectious titers of the described SARS-CoV-2 non-VOC and VOCs is provided in Table 1.

**Table 1:** Summery of peak values and time points of SARS-CoV-2 non-VOC and VOC replication kinetics in Vero E6, Caco-2, and Calu-3

	Max RNA level [copy number/mL]	Time point of max RNA level [h p.i.]	Max viral infectious titer [TCID50/mL]	Time point of max viral infectious titer [h p.i.]	Time point of visible CPE [h p.i.]
*Vero E6*					
Non-VOC	2.85E+09	96	9.17E+07	56	56
Alpha	1.70E+09	96	1.33E+07	72	56
Beta	2.98E+09	96	3.43E+06	80	80
Gamma	2.20E+09	96	2.40E+07	56	56
Delta	2.11E+09	96	3.63E+06	72	56
Omicron	2.14E+09	96	6.30E+05	96	72
*Caco-2*					
Non-VOC	4.36E+08	96	2.29E+07	96	–
Alpha	4.33E+08	96	1.08E+06	96	–
Beta	1.85E+08	96	1.52E+05	96	–
Gamma	9.30E+08	96	2.06E+06	96	–
Delta	5.86E++08	96	7.87E+05	72	–
Omicron	8.20E+05	96	3.54E+02	96	–
*Calu-3*					
Non-VOC	2.60E+08	96	1.20E+07	80	80
Alpha	1.24E+09	96	5.57E+06	96	72
Beta	1.07E+08	96	1.12E+06	80	96
Gamma	2.67E+09	96	5.16E+06	96	56
Delta	2.48E+09	96	1.98E+06	80	56
Omicron	1.28E+09	96	4.27E+06	96	80

Max, maximum; h p.i., hours post infection; CPE, cytopathic effect

## Discussion

In this alarming pandemic situation, research on SARS-CoV-2 is arising all around the globe. The main model for basic virus research is the cell culture model, using permissive cell lines to isolate, propagate, characterise and challenge the virus in question with a variety of well-known or upcoming new methods and substances. We compared viral replication kinetics of the different SARS-CoV-2 VOCs on three common cell culture models during low MOI/multi-step growth curves. It has to be noted that in contrast to high MOI/one-step growth curves, our experimental design does not provide quantitative data on defined steps of the viral replication cycle. It rather reflects successive rounds of viral replication, as they take place during isolation and propagation experiments. While this does not allow to draw conclusions on the mechanistic differences between the variants, it provides a guide to handling of the virus variants in standard cell culture models.

One of the most widely used cell lines for working with viruses in general is the African Green Monkey-derived Vero E6 kidney cell line [23, 24]. It was also the most commonly used cell line for isolating and propagating the newly emerging virus SARS-CoV-2 in late December 2019 and early 2020. Soon a vivid discussion arose about a certain selective pressure on SARS-CoV-2 when being propagated in Vero E6 cells, causing mutations mainly in the furin cleavage site of the virus’ genome during its adaptation to the lack of human TMPRSS2 in these cells [27, 42, 43]. The published data is not consistent so far: there is data stating that these cell line-adaptive mutations occur already after one or two passages on Vero E6 [42, 44] and that these mutations do not increase fitness in Vero E6 [45] but decrease fitness in human cell lines [46]. On the other hand, there are publications in line with our own observations not detecting these described mutations regarding the furin cleavage site while propagating SARS-CoV-2 on Vero E6 for four [24] or even ten passages [47]. Although the exact reason for these irregularly observed mutations is yet under debate, it has been suggested that using a genetically modified TMPRSS2-expressing Vero E6 cell line for virus isolation and propagation might avoid this problem [27, 48].

The expression levels of the two cellular entry factors ACE2 and TMPRSS2 are of great importance for cell culture models permissive for SARS-CoV-2. For cell infection, SARS-CoV-2 utilises the cellular ACE2 receptor with a limited correlation between receptor expression and permissiveness of the cell line [49]. In common with SARS- and Middle East respiratory syndrome (MERS)-CoV, SARS-CoV-2 infection is enhanced by TMPRSS2 [27]. Thus, the percentage of successfully infected cells differ between SARS-CoV-2 permissive cell lines, hence causing alterations in replication kinetics of the virus and its different variants in different model cell lines [36].

In our experiments with Vero E6 infection, the time point of reaching the highest infectivity correlated well with the visual appearance of CPE for all SARS-CoV-2 variants. This late CPE development, occurring only after the strongest increase in infectious viral particles in the supernatant, strongly supports the hypothesis that the virus is mainly released from Vero E6 cells by exocytosis instead of cell lysis [50]. This exocytosis is stated to be very efficient for SARS-CoV-2, as extracellular and intracellular virus levels were shown to be very similar [24, 36]. The sustained levels of viral RNA detected in the cellular supernatant, even after the highest infectious viral titer has been reached and has started to decline, indicates the possibility that spike-deficient particles and therefore defective and non-infective viral particles were released at these time points [23, 50].

In contrast to Vero E6 and Calu-3 cells, no visually detectable CPE caused by SARS-CoV-2 infection could be detected on Caco-2 cells despite increasing RNA copy numbers and viral titers (Additional file 3: Fig. S2). SARS-CoV-2-infected Caco-2 cells can be propagated for several weeks without showing any characteristic CPE or cell death (data not shown). This phenomenon is not completely new for Caco-2, as it was already shown in some studies for SARS-CoV-2 [41], SARS-CoV, and other virus infections [51–53].

In both human cell culture models, Caco-2 and Calu-3, the two variants with clinically most severe disease courses, Gamma and Delta [54, 55], seemed to replicate faster and to higher infectious titers than the other variants, except the non-VOC. However, individual replication kinetics of the non-VOC and the five VOCs on the three different cell lines infected do not fully represent the competition between variants within the human population [35]. Cell culture models are too distant from the human organism to draw reliable conclusions for infectiveness in humans and disease severity based only on replication kinetic experiments. This limited transferability of cell culture experiments to the epidemiologic situation is emphasized by the most recent findings regarding the newest VOC Omicron: while Omicron is rapidly spreading around the world and very efficiently infecting patients, it seems to have some replication disadvantages in the human cell lines Caco-2 and Calu-3 [56, 57]. Relative to the previously dominant variant Delta, its replication in cell culture was much slower in our experiments (Figs. 2 and 3). RNA copy numbers in the supernatant as well as infective viral titers were lagging far behind compared to the other variants. Others working with Omicron in cell culture [57, 58] or animal models [59] already observed this replication attenuation. First mechanistic investigations reveal that the Omicron variant is relatively inefficient in using TMPRSS2 in comparison to Delta and previous variants. Cell culture experiments show that Omicron is therefore less fusogenic than Delta, which may explain its reduced replication in Caco-2 and Calu-3 cells. Although the spike protein of Omicron is cleaved into two subunits, which facilitates cell–cell fusion, the spike of Omicron is less efficiently cleaved compared to Delta [58, 60, 61]. Further investigation showed that Omicron viruses are less effective than Delta viruses in antagonizing the interferon response in human cells, which may contribute to the less efficient replication in human cell lines and the lower pathogenicity of the Omicron variant observed in patients. Notably, SARS-CoV-2 proteins known to inhibit the host cell interferon response including NSP3, NSP6, NSP14, nucleocapsid, membrane, and spike protein are mutated in the Omicron variant [56].

## Conclusion

In conclusion, our study is the first systematic comparison of all SARS-CoV-2 VOCs on three different cell culture models. It shows distinct differences in viral replication kinetics of the SARS-CoV-2 non-VOC and five VOCs on the three cell culture models Vero E6, Caco-2, and Calu-3. We hope that this data provides support for researchers worldwide in their experimental design for work on SARS-CoV-2 such as performing virus isolation and propagation on Vero E6 while conducting infection studies or drug screening and antibody-based assays rather on the human cell lines Caco-2 and Calu-3.

## Supplementary Information

**Additional file 1**. SNP analysis cell line authentication report.

**Additional file 2: Fig. S1**. Progression of CPE on Vero E6. Cells were infected at an MOI of 0.0001 for 96 h. Images were taken at all time points from 2 to 96 h p.i.. For 72, 80, and 96 h p.i., representative images of SARS-CoV-2 non-VOC, the five VOCs, and an uninfected control are shown to illustrate the difference in CPE development and progression between different virus strains. CPE was considered as: (–) no CPE, (+) emerging CPE, (++) intermediate CPE, (+++) strong CPE, as indicated in the upper right corner of every microscopy picture.

**Additional file 3: Fig. S2**. Absence of CPE on Caco-2 Cells were infected at an MOI of 0.0001 for 96 h. Images were taken at all time points from 2 to 96 h p.i.. For 72, 80, and 96 h p.i., representative images of SARS-CoV-2 non-VOC, the five VOCs, and an uninfected control are shown to illustrate the difference in CPE development and progression between different virus strains. CPE was considered as: (–) no CPE, (+) emerging CPE, (++) intermediate CPE, (+++) strong CPE, as indicated in the upper right corner of every microscopy picture.

**Additional file 4: Fig. S3.** Progression of CPE on Calu-3. Cells were infected at an MOI of 0.0001 for 96 h. Images were taken at all time points from 2 to 96 h p.i.. For 72, 80, and 96 h p.i., representative images of SARS-CoV-2 non-VOC, the five VOCs, and an uninfected control are shown to illustrate the difference in CPE development and progression between different virus strains. CPE was considered as: (–) no CPE, (+) emerging CPE, (++) intermediate CPE, (+++) strong CPE, as indicated in the upper right corner of every microscopy picture.

## Data Availability

All data generated or analysed during this study are included in this published article and its supplementary information files. SNP analysis cell line authentication report is provided as Additional file 1. Consensus sequences of the viral lineages determined by WGS were submitted to the GISAID database (www.gisaid.org) with the following accession numbers: non-VOC/B.1.1 passage 1 (EPI_ISL_10201350), passage 3 (EPI_ISL_10201358), passage 4 replicate I (EPI_ISL_10201359), passage 4 replicate II (EPI_ISL_10201360), passage 4 replicate III (EPI_ISL_10201362), Alpha/B.1.1.7 patient sample (EPI_ISL_1143971), passage 2 (EPI_ISL_10201372), passage 3 (EPI_ISL_10201380), passage 4 replicate I (EPI_ISL_10201369), passage 4 replicate II (EPI_ISL_10201370), passage 4 replicate III (EPI_ISL_10201371), Beta/B.1.351 patient sample (EPI_ISL_10201379), passage 4 (EPI_ISL_10201351), passage 5 replicate I (EPI_ISL_10201352), passage 5 replicate II (EPI_ISL_10201353), passage 5 replicate III (EPI_ISL_10201354), Gamma/P.1 patient sample (EPI_ISL_1349034), passage 2 (EPI_ISL_10201363), passage 3 (EPI_ISL_10201364), passage 4 replicate I (EPI_ISL_10201365), passage 4 replicate II (EPI_ISL_10201366), passage 4 replicate III (EPI_ISL_10201367), Delta/B.1.617.2 patient sample (EPI_ISL_2260430), passage 2 (EPI_ISL_10201368), passage 3 replicate I (EPI_ISL_10201377), passage 3 replicate II (EPI_ISL_10201375), passage 3 replicate III (EPI_ISL_10201376), and Omicron/B.1.1.529 patient sample (EPI_ISL_8379763), passage 1 (EPI_ISL_10201373), passage 2 (EPI_ISL_10201378), passage 3 replicate I (EPI_ISL_10201355), passage 3 replicate II (EPI_ISL_10201356), passage 3 replicate III (EPI_ISL_10201357).

## Abbreviations

ACE2: Angiotensin-converting enzyme 2
Covid-19: Coronavirus disease 2019
CPE: Cytopathic effect
LOD: Limit of detection
MERS: Middle East respiratory syndrome
MOI: Multiplicity of infection
p.i.: Post infection
RBD: Receptor-binding domain
RT-ddPCR: Reverse transcriptase droplet digital PCR
RT-qPCR: Quantitative reverse transcription real-time polymerase chain reaction
SARS-CoV-2: Severe Acute Respiratory Syndrome Coronavirus type 2
TCID50: 50% Tissue culture infectious dose
TMPRSS2: Transmembrane protease serine subtype 2
VOC: Variant of concern
VUI: Variant under investigation
WGS: Whole genome sequencing
WHO: World Health Organization
