# Supplementary material for: Replication kinetics and infectivity of SARS-CoV-2 variants of concern in common cell culture models
Source: Virol J. 2022 Apr 26;19:76. doi: 10.1186/s12985-022-01802-5 (PMC9038516; doi:10.1186/s12985-022-01802-5)
Supplement: Supplementary file 1 — Additional file 1. SNP analysis cell line authentication report. [file 12985_2022_1802_MOESM1_ESM.pdf]

MULTIPLEXION

Landesamt für Gesundheit und Lebensmittelsicherheit
Lena Mautner

Veterinärstraße 2

85764 Oberschleißheim

Multiplexion GmbH
Virchowstr. 51
88048 Friedrichshafen
Fon: +49 (0) 7545 7759044
E-Mail: info@multiplexion.de
www.multiplexion.com

Human Cell Line Authentication - Certificate

Report ID	3391	Order ID	3549
Report Date	22.12.2021	Order Date	08.12.2021
		Purchase No.	

Dear Lena Mautner,

Many thanks for your order. The Multiplex human Cell line Authentication Test (MCA) was performed as described at www.multiplexion.de. Please find below the results.

Best regards,

Dr. Markus Schmitt

Information from Customer				Results			Summary			
Sample ID	Sample Name	Cell line name	If other: exact name	DNA quality	Best Hit with DataBase	Identity (%)	Present in Database?	Cross-Contamin-ation?	Identity confirmed?	Genotype Code
7365	Caco-2	CACO-2		ok	CACO-2	100	yes	no	confirmed	AATTTTTTAAAAAAAAATATAAATTTTTA AAAAAATATTTAAATATAA
7366	Calu-3	Calu-3		ok	Calu-3	100	yes	no	confirmed	ATTTAAAAAAAAWAAAAAAATTAATTAAT TAAAAATTTTTTTAAAAAWTT

Legend:

DNA quality: ok, good DNA quality detected; invalid, DNA was absent or degraded or from non-human species

Identity (%) Identity of submitted cell line to best hit of data base; identical: 96% and above, not indentical: <96. Please note, a 96% or 98% identity may hint at loss of heterozygosity, which might be due to overlong passaging.

Present in database? indicates whether submitted cell line is included in MCA data base. If not included, than no identity confirmation can be made.

Cross-contamination? indicates whether detected cell line is cross-contaminated by additional cells from another human cell line, the contaminating cell line cannot be specifically identified. For polyploid cell lines, cross-contamination cannot be reliably determined.

Identity confirmed? "confirmed", identity was confirmed by MCA (>95%); "false", submitted cell line shows a cross-match with a cell line present in data base; "unique sequence", cell line is not present in data base and shows a genotype code that is not related to any cell line included in the data base (<95%)

Genotype Code 48-letter code for 24 SNP locations; W, uncertain signal; N, no call
