# Supplementary figures and images for: Replication kinetics and infectivity of SARS-CoV-2 variants of concern in common cell culture models
Source: Virol J. 2022 Apr 26;19:76. doi: 10.1186/s12985-022-01802-5 (PMC9038516; doi:10.1186/s12985-022-01802-5)

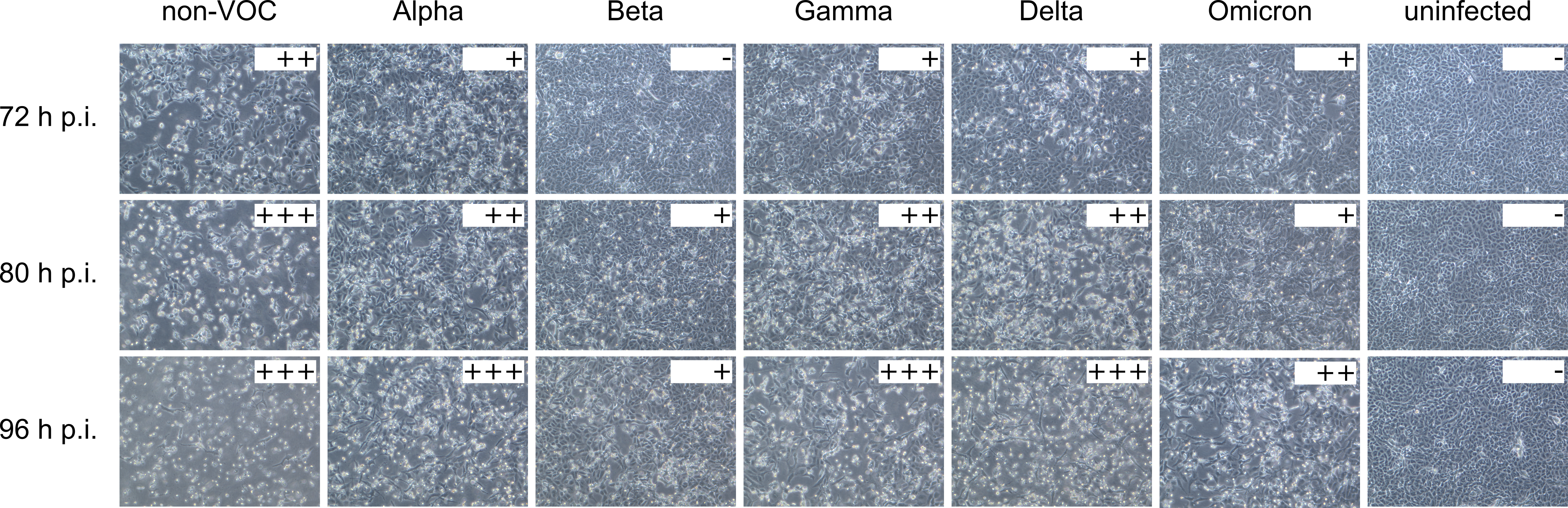

Supplement: Supplementary file 2 — Additional file 2: Fig. S1. Progression of CPE on Vero E6. Cells were infected at an MOI of 0.0001 for 96 h. Images were taken at all time points from 2 to 96 h p.i.. For 72, 80, and 96 h p.i., representative images of SARS-CoV-2 non-VOC, the five VOCs, and an uninfected control are shown to illustrate the difference in CPE development and progression between different virus strains. CPE was considered as: (–) no CPE, (+) emerging CPE, (++) intermediate CPE, (+++) strong CPE, as indicated in the upper right corner of every microscopy picture. [file 12985_2022_1802_MOESM2_ESM.png]

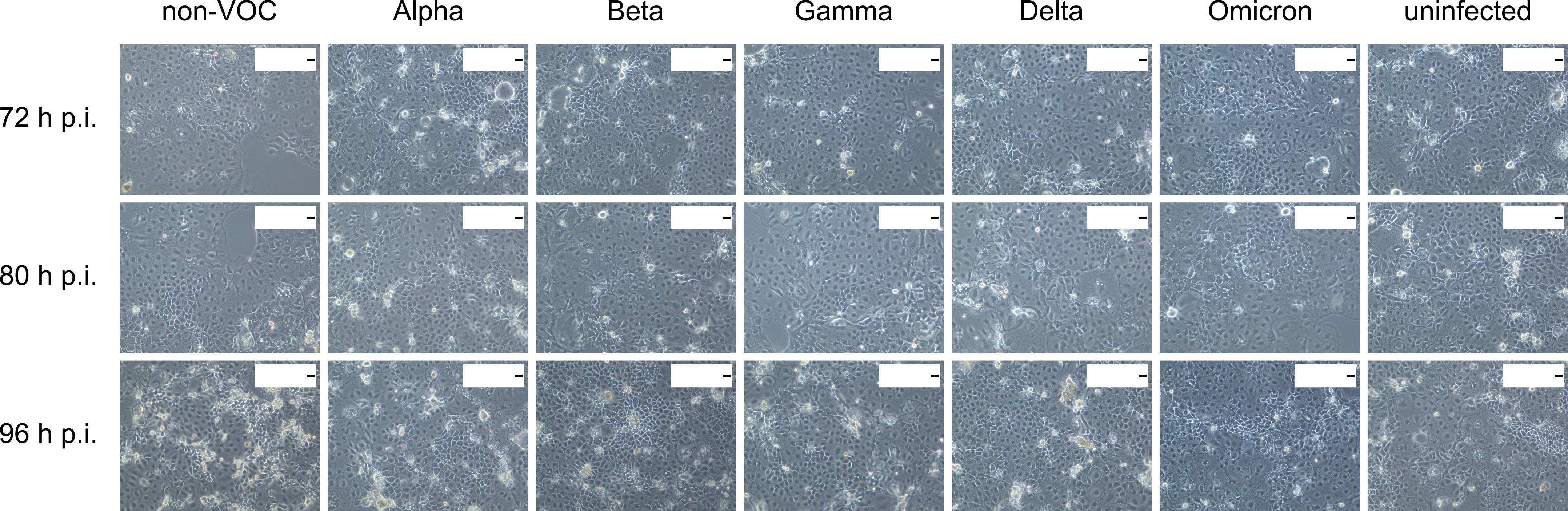

Supplement: Supplementary file 3 — Additional file 3: Fig. S2. Absence of CPE on Caco-2 Cells were infected at an MOI of 0.0001 for 96 h. Images were taken at all time points from 2 to 96 h p.i.. For 72, 80, and 96 h p.i., representative images of SARS-CoV-2 non-VOC, the five VOCs, and an uninfected control are shown to illustrate the difference in CPE development and progression between different virus strains. CPE was considered as: (–) no CPE, (+) emerging CPE, (++) intermediate CPE, (+++) strong CPE, as indicated in the upper right corner of every microscopy picture. [file 12985_2022_1802_MOESM3_ESM.png]

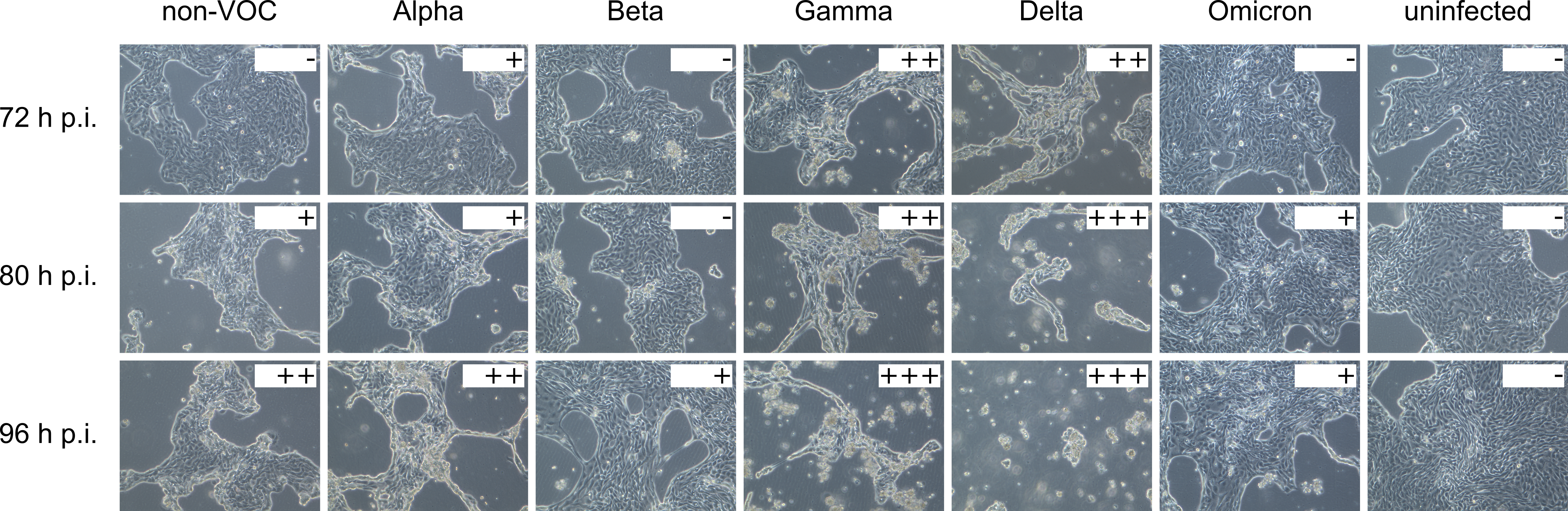

Supplement: Supplementary file 4 — Additional file 4: Fig. S3. Progression of CPE on Calu-3. Cells were infected at an MOI of 0.0001 for 96 h. Images were taken at all time points from 2 to 96 h p.i.. For 72, 80, and 96 h p.i., representative images of SARS-CoV-2 non-VOC, the five VOCs, and an uninfected control are shown to illustrate the difference in CPE development and progression between different virus strains. CPE was considered as: (–) no CPE, (+) emerging CPE, (++) intermediate CPE, (+++) strong CPE, as indicated in the upper right corner of every microscopy picture. [file 12985_2022_1802_MOESM4_ESM.png]
